# Quantification of Contractile Dynamic Complexities Exhibited by Human Stem Cell-Derived Cardiomyocytes Using Nonlinear Dimensional Analysis

**DOI:** 10.1038/s41598-019-51197-7

**Published:** 2019-10-11

**Authors:** Plansky Hoang, Sabir Jacquir, Stephanie Lemus, Zhen Ma

**Affiliations:** 1 Department of Biomedical and Chemical Engineering, Syracuse University, Syracuse, NY USA; 2 Syracuse Biomaterials Institute, Syracuse University, Syracuse, NY USA; 3 Department of Integrative and Computational Neuroscience, Paris-Saclay Institute of Neurosciences, Université Paris-Sud - Université Paris-Saclay, Cedex, France

**Keywords:** Biological techniques, Stem cells, Heart stem cells

## Abstract

Understanding the complexity of biological signals has been gaining widespread attention due to increasing knowledge on the nonlinearity that exists in these systems. Cardiac signals are known to exhibit highly complex dynamics, consisting of high degrees of interdependency that regulate the cardiac contractile functions. These regulatory mechanisms are important to understand for the development of novel *in vitro* cardiac systems, especially with the exponential growth in deriving cardiac tissue directly from human induced pluripotent stem cells (hiPSCs). This work describes a unique analytical approach that integrates linear amplitude and frequency analysis of physical cardiac contraction, with nonlinear analysis of the contraction signals to measure the signals’ complexity. We generated contraction motion waveforms reflecting the physical contraction of hiPSC-derived cardiomyocytes (hiPSC-CMs) and implemented these signals to nonlinear analysis to compute the capacity and correlation dimensions. These parameters allowed us to characterize the dynamics of the cardiac signals when reconstructed into a phase space and provided a measure of signal complexity to supplement contractile physiology data. Thus, we applied this approach to evaluate drug response and observed that relationships between contractile physiology and dynamic complexity were unique to each tested drug. This illustrated the applicability of this approach in not only characterization of cardiac signals, but also monitoring and diagnostics of cardiac health in response to external stress.

## Introduction

Over recent years, there has been increasing interest of using nonlinear analysis to study human biological signals, since maintenance of stability in physiological functions involves interconnected feedback loops between biological systems. Such high degrees of interdependency require in-depth investigation on the level of complexity and variability of the biological systems for better understanding of their regulatory mechanisms. Specifically, cardiac rhythm has gained widespread attention, because it is known to exhibit considerable signal complexity in healthy individuals from normal day-to-day activities. Additionally, cardiovascular diseases are often associated with irregularities in cardiac contraction, frequency and rhythm^1^. Cardiac physiology is traditionally quantified using linear analytical approaches that analyze acquired physiological signals (*e*.*g*. electrocardiograms (ECG)) in the time and frequency domains^2–4^. There is increasing evidence suggesting that nonlinear analysis can be used in conjunction with standard diagnostic methods to quantify individual physiological states^5^. Differences in the nonlinear indices can be monitored between healthy and sick individuals, including cases of emotional stress, environmental changes, or genetic cardiomyopathies.

Methods from chaos theory and nonlinear dynamics give us the opportunity to study these complex biological signals, including entropy^6^, complexity indexes^7^ and dimensional analysis^8^. Phase space reconstruction (PSR)^9,10^ and dimensional analysis has been implemented to characterize the intrinsic nonlinear complexity within the time series signals^11–13^. Specifically, the phase space consists of a set of typical trajectories of the system, in which each point corresponds to one system state. The dimensionality of PSR can be quantified by determining the fractal properties of the attractors in a dynamic system based on different calculation methods, including capacity, information and correlation dimensions. The fractal is a subset of points at small scale that can resemble the whole object. The fractal properties represent the degree of complexity, to which the object differs from the Euclidean geometry with integer topological dimensions. Analyzing the fractal properties of a waveform represents a useful tool for identifying the number of independent variables necessary for generating a corresponding process or state.

Despite the application of nonlinear analysis to the clinically acquired signals (*e*.*g*. ECG), such analysis has not been widely applied for *in vitro* cardiac model systems. With the rising interest in human induced pluripotent stem cell (hiPSC) derived cardiomyocytes (hiPSC-CMs), it is of utmost importance that we are able to characterize their contractile functions and heartbeat rhythm in a more precise and comprehensive manner. However, current approaches to characterize the contractile physiology of hiPSC-CMs still heavily depend on amplitude^3^ and frequency^2^ analyses of physical and electrophysiological data. These conventional analytical tools only draw simplified readouts from complex biological signals, which poses the challenge of gaining informative analytical outcomes from hiPSC-CMs to assess comparability with native tissue and responsiveness to external stress^14^. In addition, hiPSC-CMs exhibit higher variability^15,16^ in their contractile behaviors than the adult human heart even under normal conditions, which further emphasizes the need to integrate novel analytical methods to *in vitro* cardiac model systems.

In this work, we have introduced a novel analytical tool that performs nonlinear dimensional analysis on the contractile dynamics of hiPSC-CMs. More specifically, we used optical flow analysis based on block matching to detect cardiac contractile motions of hiPSC-CMs within a video, generate contractile motion waveform in the time domain, and perform conventional amplitude and frequency analyses. Then, we implemented PSR to the motion waveforms to derive dimensional parameters that will quantify the complexity of contractile motion signals. To assess the applicability of this new analytical approach, we applied this analytical workflow to analyze the contractile complexity of hiPSC-CMs exposed to various external stressors, including drug exposure and electrical stimulation. We envisage that this analytical tool can be complemented to the rapid expanding field of *in vitro* cardiac tissue model by quantifying the irregularity and complexity of cardiac contractile motions, which have been difficult to answer via conventional methods. This approach also will provide new insights on how chaotic theory and nonlinear dynamics can be used for *in vitro* biological experimental models.

## Materials and Methods

### hiPSC culture and differentiation

Wild-type (WT) hiPSCs were grown on hESC-qualified growth factor-reduced Geltrex (Life Technologies) coated substrates and maintained in Essential 8 medium (E8) (Life Technologies). Differentiation of hiPSC-CMs were performed based on the protocol that uses two small molecules to modulate canonical WNT pathway^17^. hiPSC-CMs were maintained in RPMI basal medium supplemented with B27 complete (RPMI + B27-C), and medium was changed every two days. After 12 days culturing in RPMI + B27-C, hiPSC-CMs were dissociated, singularized and replated for purification procedures with glucose-depleted lactate medium^18^.

### Drug exposure

To evaluate drug response, hiPSC-CMs were treated for both long and short terms to the drugs affecting the heart rate. For long term exposure, hiPSC-CMs were treated with a constant 10 nM isoproterenol in RPMI + B27-C for 1 week. The drug-supplemented medium was changed every two days and videos were recorded daily. For short term exposure, hiPSC-CMs were treated with three drugs (alfuzosin, flecainide and isoproterenol) at two doses and videos were recorded 10 minutes after each dose. Doses were increased by adding the appropriate volume of stock solution to the hiPSC-CM culture medium.

### Electrical stimulation

hiPSC-CMs were electrical stimulated using a C-Pace unit and a 6-well C-Dish, according to the manufacturer manual (Ion Optix, Milton, MA, USA). The carbon electrodes supplied with rectangular, 5 V/cm, 2 ms and 2 Hz electrical pulses to the hiPSC-CMs. The baseline beating videos were recorded as *Pre Stim* before the electrical stimulation was supplied, and then hiPSC-CMs were stimulated for 30 minutes as the conditioning stimulation. Next, three beating videos were recorded from the hiPSC-CMs under electrical stimulation as *ON 2* *Hz Stim* with 30-minute time intervals between two videos. Last, 2 hours after the stimulation was removed, the beating videos of the hiPSC-CMs were recorded as *Post Stim*.

### Video recording and motion-tracking analysis

hiPSC-CMs were imaged in an onstage microscope incubator at 37 °C and 5% CO_2_ to maintain standard physiological conditions on a Nikon Ti-E inverted microscope with Andor Zyla 4.2+ digital CMOS camera. Videos of the beating hiPSC-CMs were recorded at 100 fps over 20 seconds in bright-field, and exported as a series of single-frame image files. The image series were then analyzed using in-house and open source motion tracking software^19^ that calculates motion vectors based on block matching of macroblocks of pixels from one frame to the next. The software then generates motion waveforms that represent the contractile physiology of hiPSC-CMs.

### Calculation of the capacity dimension

The capacity dimension of contraction motion waveforms (Supplemental Fig. 1a) was calculated using the variogram method based on a “variation estimator”^20^. The variation estimator plots the variance γ (*τ*) of values given for the points separated by a certain distance *τ*, given by

1 $$\gamma (\tau )=\frac{1}{2(N-\tau )}\mathop{\sum }\limits_{i=\tau }^{N}{[|{S}_{(i+1:N)}-{S}_{(1:N-i)}|]}^{2}$$

where N is the number of neighboring data points within the time lag distance specified, *S*_*i*+*1:N*_ is the value of the initial point, and *S*_*1:N*−*i*_ is the value of the neighboring point being compared in the range of *i* bounded by the time lag distance *τ* and the length *N* of the contraction motion waveform *S*. Thus, for computing capacity dimension using variogram approach, first step is to compute the variance γ(*τ*) for different time lag *τ*. This can be plotted as a curve, in which the variogram value increases with time lag to a maximum, and levels off at a time lag where the total variability of the data field is reached. Next, log(γ(*τ*)) *vs*. log(*τ*) is plotted, and regression method is used to calculate the slope *P* of the line. Finally, the capacity dimension is given by

2 $${D}_{capacity}=2-P/2$$

### Calculation of the correlation dimension

The correlation dimension (*D*_*correlation*_) measures the geometrical complexity of an attractor^21^ and has become a standard measure of the fractal properties of attractors that have been reconstructed in the state space. A larger value of the correlation dimension depicts a higher degree of complexity in the signal dynamics. If the dynamics is stochastic, *D* tends to infinity and the attractor is obtained when the signal is embedded in a phase space. From a cardiac contractile motion waveform (*S*_*n*_; n = 1, …, N) with N samples, considering Takens theorem^22^, an m-dimensional phase-space is constructed as follows:

3 $$\begin{array}{c}\overrightarrow{{x}_{j}}=({S}_{j},\,{S}_{j+\tau },\,{S}_{j+2\tau },\ldots ,{S}_{j+(m-1)\tau })\\ \,\,\,\,\,\,\,\,\,j=1,\ldots ,\,N-(m-1)\tau \end{array}$$

The phase space reconstruction depends on two parameters: time lag *τ* and embedding dimension *m*. If τ is too small, the trajectories of S_j_ and S_j+τ_ are too close to be separated. In contrast, if τ is too large, the trajectories of attractor projected on the two axes are not correlated, which makes the reconstructed phase space useless. The goal is to find the smallest value of τ to ensure the independence of resulted coordinates in the phase space. Herein, the time lag τ is computed using the method of autocorrelation function (ACF)^23^, and it corresponds to the time required for the ACF to decrease to 1/*e* of its original value as follows:

4 $$C(\tau )=C(0){e}^{-k\tau }$$

where *C(0)* is calculated without a time lag (τ = 0). τ can be defined when $C(\tau )=\frac{C(0)}{e}$ (where *k* = *1/τ*), which corresponds to the time required for the ACF to decrease to 1/*e* of its original value.

In terms of the embedding dimension *m*, a well-defined embedding dimension is essential to a phase space to describe all possible states of a dynamic system. Since the dimension of the phase space reconstructed from experimental data is not known in priori, embedding dimension *m* will be determined to ensure that reconstructed phase space is topologically identical to original data. From a geometrical point of view, the time series (experimental data) is the projection of a *m*-dimensional system (reconstructed phase space) to a one-dimensional space. Therefore, two points in the *m*-dimensional space, even far from each other, could be very close in the original 1D space, which makes them false neighbors. In the reconstructed phase space, the distances between a point and its nearest neighbor will be measured. If two points are real nearest neighbors, the distance will not change with the increase of dimension. Herein, the embedding dimension *m* is estimated using the method of False Nearest Neighbors (FNN)^24^, where the optimal value of *m* corresponds to the minimum value of *m* for which the FNN is close to zero.

In our work, the correlation dimension is determined using the Grassberger-Procaccia method^25^, based on the following approximation: the probability of having a couple of points in a box of size *r* is equal to the probability of having a couple of points with separation distance less than *r* when $r\to 0$. The correlation dimension is defined by:

5 $${D}_{correlation}=\mathop{\mathrm{lim}}\limits_{r\to 0}[\frac{\log ({{\rm{C}}}_{{\rm{m}}}(r))}{\log (r)}]$$

where the correlation integral C_m_(r) is approximately given by:

6 $${C}_{m}(r)\approx \frac{2}{N(N-1)}\mathop{\sum }\limits_{j=1,\,i > j}^{N}\varTheta (r-\parallel {x}_{j}-{x}_{i}\parallel )$$

where Θ(x) is the Heaviside step function. The summation counts the number of pairs (x_i_ x_j_) for which the distance || x_i_, x_j_ || is less than *r*. For each reconstructed phase space trajectory, the distances between all points in the trajectory are calculated. The logarithm of the smallest distance (represented by *r*_*min*_) and the logarithm of the largest distance (represented by *r*_*max*_) are then computed. A series of bins is created to record the correlation sum, *C*_*m*_*(r)*, which is the normalized number of couples of points with a separation distance less than a specified distance *r*. The process of depositing counts of data into bins is analogous to recording counts of the occurrence of events within data in a frequency histogram. In this study, an arbitrary number of 32 bins is used and the width of each bin is set to $\frac{{r}_{max}-{r}_{min}}{32}$. Thus, from first to last, the separation distances ${r}_{n}={r}_{min}+\frac{n({r}_{max}-{r}_{min})}{32}$, where *n* = 1 to 32 are considered. In practice, the correlation dimension is obtained from the slope of log (*C*_*m*_(*r*)) versus log (*r*). Several *C*_*m*_*(r)* are computed for increasing values of the embedding dimension *m*, and the slopes are determined from a scaling region of the log-log plot, as shown in Supplemental Fig. 1b. When *m* increases, *D*_*correlation*_ reaches to a saturation value corresponding to a steady correlation dimension^26,27^ (Supplemental Fig. 1c). From the distribution of the correlation dimension values (Supplemental Fig. 1d), the averaged value of the correlation dimension of this set is computed. Based on the curvature of *D*_*correlation*_ values, the steady correlation dimension corresponding to the curve plateau was computed for each set of *D*_*correlation*_, illustrated in Supplemental Fig. 1c.

### Statistical analysis

Data was plotted as the mean ± s.d. For single comparisons between groups, a two-sided Student’s t-test was used, and *p* ≤ *0*.*05* was considered significant.

## Results

### Nonlinear dimensional analysis on contractile motions

**Figure 1:**
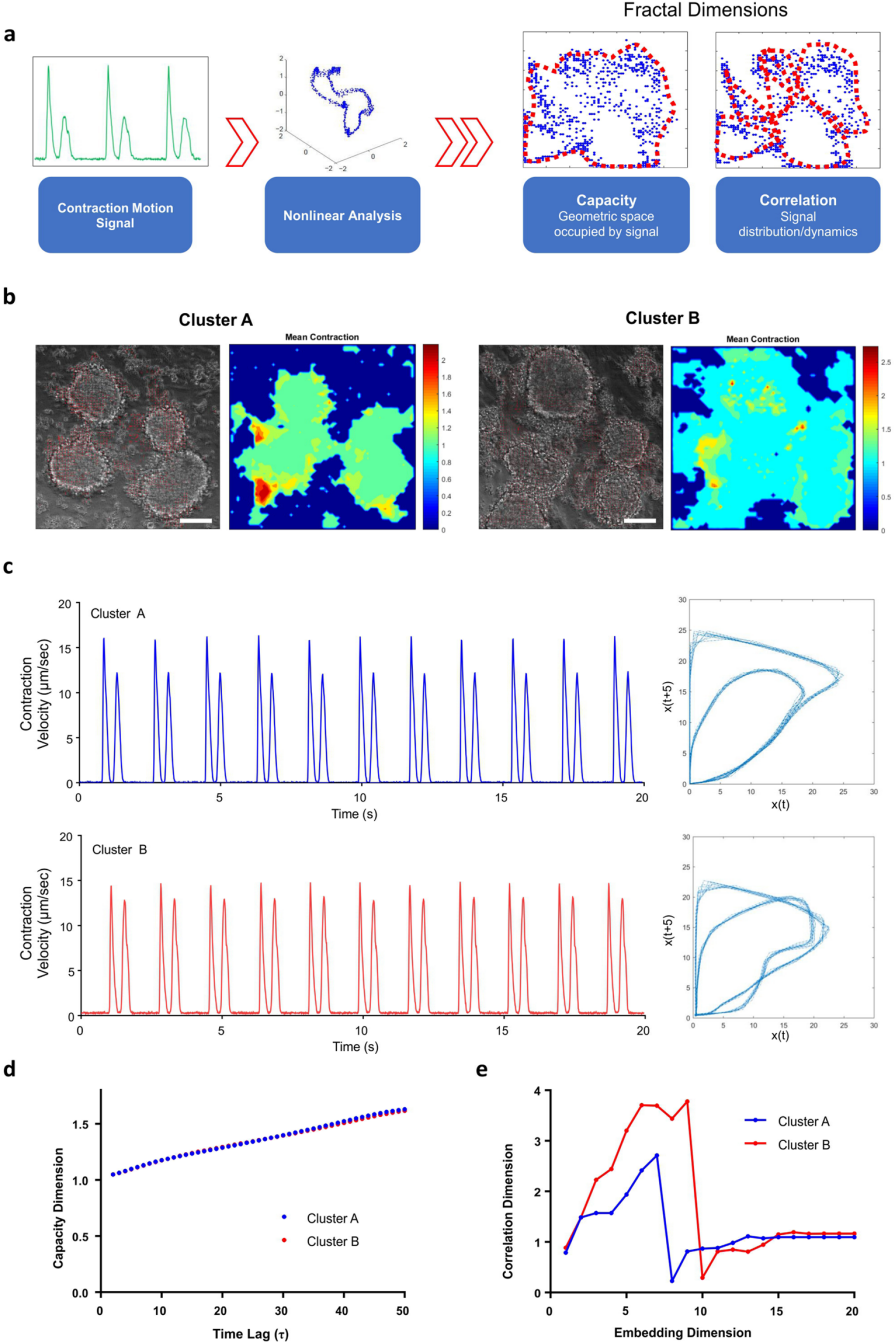
Nonlinear reconstruction of time series contractile motion signals. (**a**) Contraction motion waveforms were analyzed using phase space reconstruction to determine the fractal properties of the signals, including two parameters: capacity dimension and correlation dimension, which characterize the geometric space and distribution dynamics of the signals. (**b**) Two hiPSC-CM clusters (*Cluster A and Cluster B*) exhibited comparable contractile physiology, (**c**) which were compared in time domain and respective nonlinear phase spaces. (**d**) The capacity dimension with respect to time lag (τ) was comparable between the two clusters, (**e**) but the correlation dimension with respect to embedding dimension (*m*) showed different trends. Scale bars = 100 μm.

In order to determine the complexity of cardiac contractile dynamics, we performed phase space reconstruction of the contractile motion waveforms recorded from hiPSC-CMs. To analyze the fractal properties of reconstructed phase space, we can compute capacity and correlation dimensions based on different methods (Fig. 1a). hiPSC-CMs were differentiated from WT hiPSCs via modulation of the WNT/β-catenin pathway and enriched using a lactate-supplemented purification method^17^. After purification, we recorded the beating videos of hiPSC-CM clusters (Fig. 1b), and compared the difference in both linear and nonlinear parameters between two independent tissue cluster samples (*Cluster A vs*. *Cluster B*). Motion waveforms corresponding to *Cluster A* and *Cluster B* were visibly comparable based on visual inspection (Fig. 1c), which was corroborated with similar motion results (Table 1), including comparable beat rates (33.17 *vs*. 33.99 beats per minute), contraction velocities (24.65 *vs*. 22.46 µm/s), and relaxation velocities (18.69 *vs*. 19.97 µm/s). Based on these two motion waveforms, the peak-to-peak (PP) interval was also computed as a traditional linear assessment. Similarly, the average PP interval remained relatively consistent between both samples (1.81 *vs*. 1.77 seconds). Therefore, from motion tracking analysis, we concluded that these two samples exhibited similar contractile features based on linear time series analysis.

**Table 1:** Comparison of linear and nonlinear parameters of Cluster A and Cluster B.

	Cluster A	Cluster B
Beat Rate (BPM)	33.17 ± 0.349	33.99 ± 0.227
Peak-Peak Interval (s)	1.81 ± 0.016	1.765 ± 0.012
Contraction Velocity (µm/sec)	16.02 ± 0.187	14.599 ± 0.143
Relaxation Velocity (µm/sec)	12.15 ± 0.075	12.98 ± 0.142
Mean Capacity Dimension	1.354	1.350
Steady Correlation Dimension	1.193	1.354

Nonlinear PSR analysis was then applied to extend the characterization of these two contraction motion waveforms (Fig. 1c). The raw data acquired from motion tracking analysis was processed using custom algorithms, from which we were able to calculate the capacity and correlation dimensions based on time lag (*τ*) and embedding dimension (*m*), respectively. The mean capacity dimension (*D*_*capacity*_) was also similar between two samples (1.354 *vs*. 1.350) (Fig. 1d), inferring that the geometrical capacity of phase space between two clusters was comparable. We also calculated and plotted the correlation dimension (*D*_*correlation*_) with respect to embedding dimension, which is a measure of the dynamic distribution of points in the phase space (Fig. 1e). In contrast, Cluster B exhibited a greater steady correlation dimension (1.193 *v*.*s*. 1.354). Therefore, the correlation dimension was able to detect the contrasts in system dynamics of the nonlinear plots between two samples exhibiting similar contractile behaviors. Computation of the correlation dimension provided quantification of additional aspect of the system dynamics, which were not visibly apparent in the motion waveforms as well as not reflected in the capacity dimension.

### Contractile variability over daily and hourly time scales

To examine natural variability of contractile physiology exhibited by hiPSC-CMs, we first recorded the beating videos and assessed the contraction motions of hiPSC-CMs daily over a course 30 consecutive days (long time scale) as well as hourly over a 7-hour time frame (short time scale). For both long and short time series, six beating tissue clusters were used for video recording and each sample was recorded once at each time point. Each beating video was analyzed for the entire field-of-view and outputted a single time series contraction motion waveform. Throughout these periods, we observed fluctuations in the average values and standard deviations of the beat rate and contraction/relaxation velocities across 6 different samples (Supplemental Fig. 2a–f). This observation demonstrated that hiPSC-CMs exhibited natural variations in their contractile behaviors over daily and hourly time scales.

By performing nonlinear analysis on the motion data, we plotted the mean capacity dimension and steady correlation dimensions for 6 samples over daily and hourly time scales (Supplemental Fig. 2g–j). Over the 30-day period, both the capacity and correlation dimensions exhibited significant fluctuations in the average values and relatively high variations across six tissue samples, which were illustrated by standard deviations for each value (Supplemental Fig. 2g,i) and daily trends for individual tissue cluster (Supplemental Fig. 3a). However, in the hourly time scale, these dimensional parameters remained relatively consistent over 7 hours (Supplemental Figs 2h,j, 3b), which implied that the hiPSC-CMs exhibited higher degrees of contraction complexity on daily basis as opposed to hourly.

**Figure 2:**
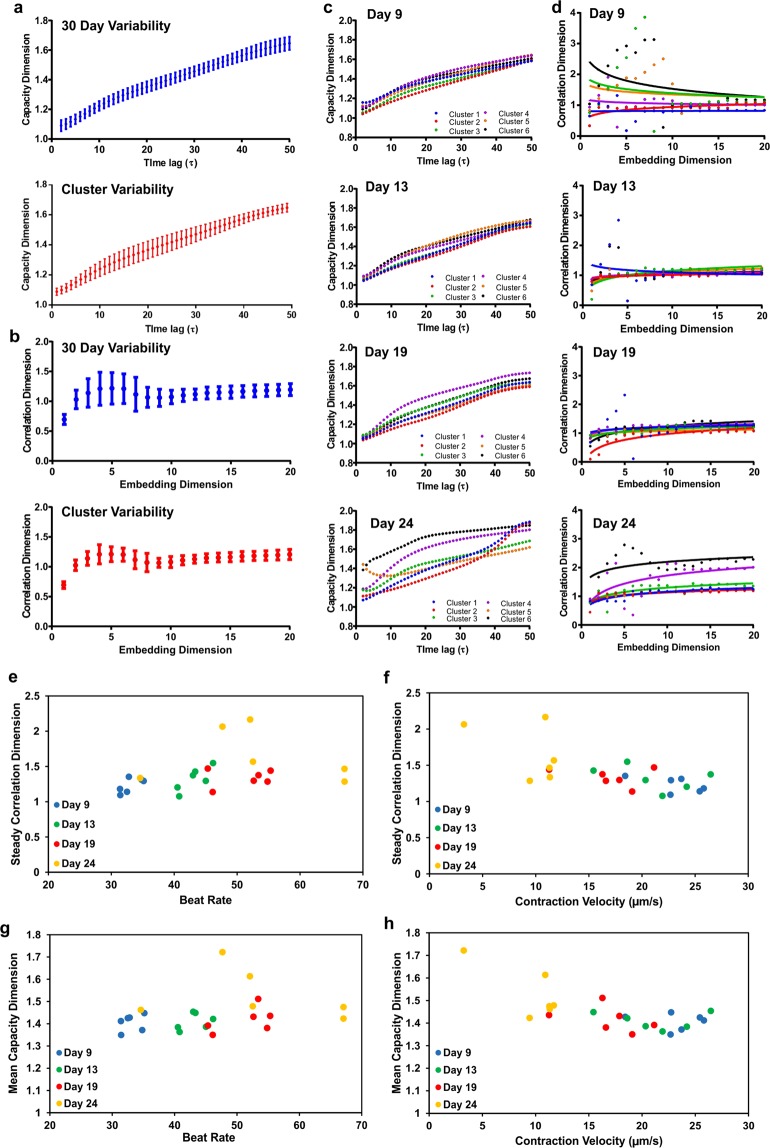
Effects of contractile physiology on nonlinear metrics. (**a**) The capacity dimension and (**b**) correlation dimension were plotted with respect to their nonlinear domains. The error bars represent the standard deviation over the 30-day period or across the six tissue clusters. Four arbitrary days were chosen for in-depth analysis (Days 9, 13, 19, and 24). (**c**) Capacity dimensions and (**d**) correlation dimensions for these particular days were plotted for each tissue cluster with respect to its’ nonlinear domain, illustrating greater fractal properties for tissues exhibiting arrhythmia-like characteristics (Day 24). (**e**,**f**) The steady correlation dimension relative to the beat rate and contraction velocities were plotted for each individual cluster on these specific days. (**g**,**h**) Similarly, the relationship between mean capacity dimensions with respect to the beat rate and contraction velocities were plotted for each tissue cluster.

We then compared the standard deviations of the nonlinear metrics reflecting the variability across the studied time periods and across the six tissue clusters. The standard deviations in capacity dimensions showed greater variations among different hiPSC-CM clusters, in contrast to the variations among different days or hours (Fig. 2a, Supplemental Fig. 3c). This implies that the overall geometric capacity of reconstructed phase space varies at a higher degree between tissue samples, because although each cluster has its unique contraction behaviors, overall contraction trends of all clusters over time appear to follow similar trends (Supplementary Fig. 3a). Contrastingly, the correlation dimensions with respect to the embedding dimension (Fig. 2b; Supplemental Fig. 3d) illustrated larger deviations across the 30 days in comparison to clusters. This might be caused by the greater variations in the signals’ dynamic distribution within the phase space over the course of 30 days. This indicated higher instances of subtle changes in the cardiac contraction, such as aberrations, that occur over the course of 30 days are more prominent in comparison to the variations among clusters. Hourly variability (Supplemental Fig. 3c) shows comparable trends between hourly and cluster deviations. Based on our previous conclusions, the comparability can be attributed to the signal variability due to the significantly shorter time scale.

To correlate the linear analysis with nonlinear metrics, we chose four arbitrary days (Day 9, Day 13, Day 19, and Day 24), and compared contractile physiology (Supplemental Fig. 4a–d) of all six clusters with their corresponding correlation and capacity dimensions. We observed that hiPSC-CMs clusters on Day 19 and Day 24 exhibited higher beat frequency than the same ones on Days 9 and Day 13. All cluster samples on Day 24, however, exhibited high degrees of irregular arrhythmia-like contractile behavior, as the motion waveforms illustrated significantly reduced contraction peaks and increased presence of signal aberrations. The increase of abnormal cardiac behavior over time was captured in the capacity dimensions, since the variations in capacity dimension curves across time lag τ are more scattered among clusters on Day 19 and particularly on Day 24 (Fig. 2c). These high variations are also illustrated in the correlation dimension, as the correlation plateaus significantly vary on Day 24, in comparison to the relatively consistent correlation plateaus of the other three days (Fig. 2d).

To understand how the nonlinear metrics are influenced by contraction amplitude and frequency, the steady correlation and mean capacity dimensions of individual clusters were plotted against the respective contraction velocity and beat rate. The steady correlation dimensions for Days 9, 13, and 19 remained relatively consistent, despite the considerable range in contraction rate and velocity across all samples (Fig. 2e,f). In contrast, clusters on Day 24 exhibited the greatest variation among different clusters in terms of contraction rate, velocity and correlation dimension. This was attributed to the irregular arrhythmia-like contractile behaviors observed from each individual cluster on this specific day. The mean capacity dimensions also remained consistent for all days with respect to beat rate and contraction velocity for all days except Day 24 (Fig. 2g,h). Overall, these associations suggest that the fractal properties of the reconstructed phase space were not intensively influenced by contraction rate and velocity averaged from time series data, but rather reflected the presence and severity of arrhythmic behaviors that contribute to the signal complexity.

### Drug response

**Figure 3:**
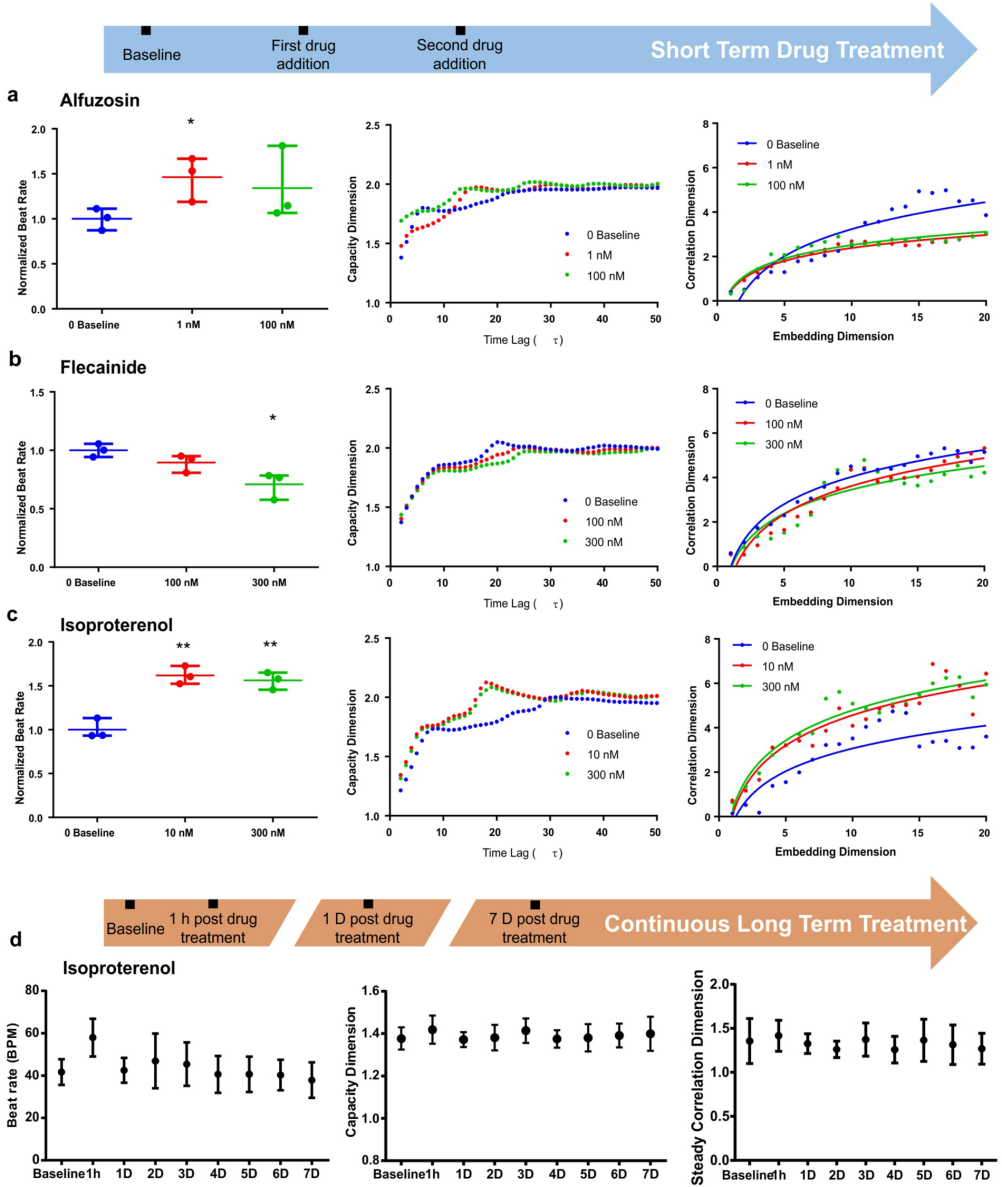
Nonlinear analysis on hiPSC-CM drug response. hiPSC-CMs was treated with 3 drugs, including (**a**) alfuzosin, (**b**) flecainide and (**c**) isoproterenol over a short period with incremental doses. The beat rate, capacity dimension and correlation dimension were compared respectively in response to the drug dosages. (**d**) A long-term drug study was also conducted by supplying a constant dose of isoproterenol over the course of 7 days.

To investigate the capability of our new analytical approach to evaluate the responsiveness of hiPSC-CMs to drug interference, hiPSC-CMs were exposed to three drug compounds (alfuzosin, flecainide and isoproterenol) known to modulate heart contractile behaviors. For this study, we recorded the beating videos for both untreated baseline controls and drug-treated hiPSC-CMs over short- and long-term drug exposure. For short-term dose-response assessment, hiPSC-CMs were treated with incremental doses of specific drugs and subsequently recorded as beating videos following each dosage. The videos were then analyzed with motion tracking to generate the corresponding waveforms (Supplemental Fig. 5a–c) and followed with nonlinear dimensional analysis. Alfuzosin is an alpha-adrenergic blocker prescribed to treat benign prostatic hyperplasia^28^. However, alfuzosin use has also shown adverse effects on prolongation of QT interval by delaying cardiac repolarization^28^. Treatment with alfuzosin significantly increased the beat rate of hiPSC-CMs (Fig. 3a), but with negligible change in average contraction velocities (Supplemental Fig. 5d). Despite the induced changes in contraction rate, nonlinear analysis showed insignificant changes in capacity dimension with increasing dose. Alternatively, the drug treatment at both the 1 nM and 100 nM concentrations resulted in an observable decrease of the correlation dimension relative to the baseline control (Fig. 3a). Next, hiPSC-CMs were treated with flecainide, which is a sodium ion channel blocker and is prescribed to restore normal heart rhythm from arrhythmias^29^. In contrast to alfuzosin, the introduction of flecainide caused a decrease in the beat rate, with slight, but insignificant increases in contraction velocities (Supplemental Fig. 5e). This corresponded to insignificant variations in capacity dimension and correlation dimension with each incremental dose (Fig. 3b). Lastly, we treated the hiPSC-CMs with isoproterenol, which is a potent β adrenergic agonist that is used clinically to treat bradycardia. hiPSC-CMs treated with isoproterenol had significantly greater beat rate and contraction velocities (Supplemental Fig. 5f), as well as higher capacity and correlation dimensions (Fig. 3c).

For the long-term drug exposure study, hiPSC-CMs were treated continuously with a constant dose (10 nM) of isoproterenol for 7 consecutive days, wherein the drug supplemented media was changed every two days and videos were recorded daily (Fig. 3d). Throughout the entire treatment period, the average contraction velocities of the tissues remained relatively consistent (Supplemental Fig. 4g,h). Regarding contraction frequency, a significant increase in beat rate was found only after the first hour of treatment. Subsequent days after, the contractile physiology returned to the baseline level, indicating desensitization of the hiPSC-CMs to the drug. The reduced sensitivity can be attributed to the fact that hiPSC-CMs are still physiologically immature with limited responsiveness to β adrenergic stimulation^30,31^. Regardless of the drug-induced changes to contractile physiology, the capacity dimension remained consistent with insignificant fluctuations, after the initial hour and over the 7-day period. In contrast, the correlation dimension exhibited significant variation only towards the last two days of treatment.

### Electrical stimulation

**Figure 4:**
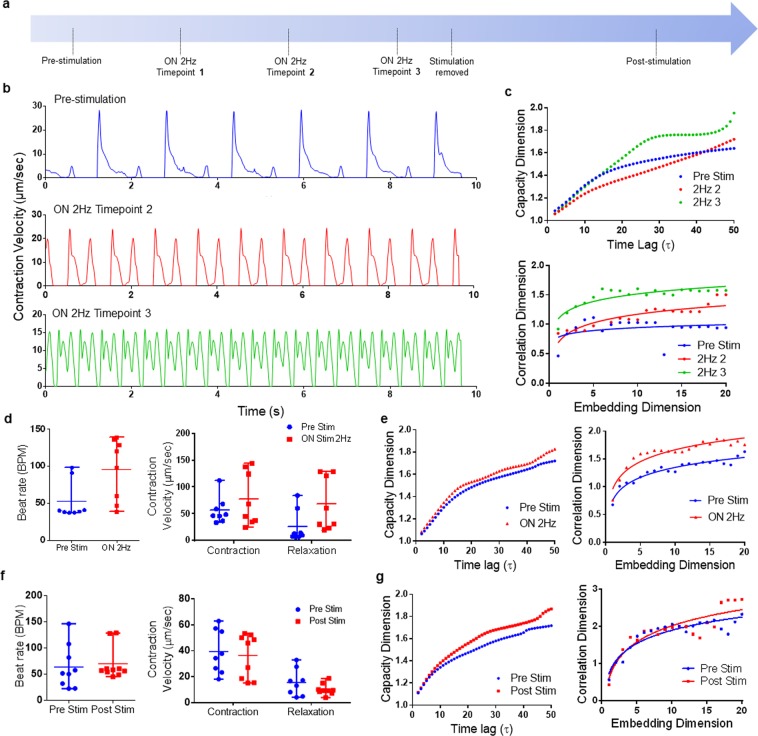
Changes in fractal properties induced by electrical stimulation. (**a**) hiPSC-CMs under electrical stimulation were recorded before (Pre Stim), during (ON 2 Hz Stim), and 2 hours after stimulation removal (Post Stim). (**b**) Corresponding motion waveforms illustrated that hiPSC-CMs were able to acquire the pacing of 2 Hz stimulatory conditions. (**c**) The stimulation induced changes in the system dynamics, as illustrated by the increases in the capacity and correlation dimensions of an individual hiPSC-CMs sample. For an average of 8 samples, ON 2 Hz Stim not only increased (**d**) beat rate, contraction and relaxation velocities, but also increased (**e**) capacity and correlation dimensions, comparing to Pre Stim condition. (**f**) Removal of stimulation (Post stim) resulted in recovery to their Pre-Stim state of beat rate, contraction and relaxation velocities. (**g**) However, the capacity and correlation dimensions of Post Stim state were still greater than the Pre Stim state.

Electrical stimulation is commonly applied to hiPSC-CMs to control intermittent pacing and promote cell maturity. We subjected hiPSC-CMs to 2 Hz stimulation and recorded videos before (*Pre Stim*), during (*ON 2* *Hz Stim*) and after stimulation removal (*Post Stim*) (Fig. 4a). During stimulation, videos were recorded with 30-minute intervals. At Timepoint 2, the hiPSC-CMs gained half the supplied pacing (60 BPM) and attained full pacing at time-point 3 (120 BPM) (Fig. 4b) as well as an overall increase in contractile physiology. We first performed individual assessments on a single sample and found that increased pacing of hiPSC-CMs was accompanied with increases in both capacity and correlation dimensions (Fig. 4c). On average for all tissue samples undergoing stimulation, the contraction rates and velocities increased, and the capacity and correlation dimensions were greater relative to their state prior to stimulation (Fig. 4d,e), indicating the changes in signal complexity and fractal characteristics due to the stimulatory conditions. This implied that nonlinear contractile dynamics was highly sensitive to the stimulatory conditions, as they significantly vary between prior and during stimulation. According to the comparison between *Pre Stim* and *Post Stim*, we observed that hiPSC-CMs returned to the baseline contractile behaviors (beat rate, contraction velocity, and relaxation velocity), resulting in no significance before and after electrical stimulations (Fig. 4f). Despite negligible change of contractile physiology motion, the nonlinear analysis showed higher values corresponding to *Post Stim* results, especially for the capacity dimension (Fig. 4g), which suggested that nonlinear dimensional analysis can possibly distinguish subtle differences underlying the stimulatory response of complex cardiac contractile physiology.

## Discussion

In this work, nonlinear dimensional analysis was supplemented to traditional linear approaches to characterize contractile dynamics exhibited by hiPSC-CMs. Specifically, we explored the variances in the fractal properties of reconstructed phase space by deriving two different parameters, capacity and correlation dimensions, which described the geometrical structure and frequency distribution of the points in the phase space reconstructed based on the contractile motion signals. We demonstrated that hiPSC-CMs with similar contractile physiology can express different nonlinear dynamics metrics, since linear analysis of contraction motion only addresses bulk average values of amplitude and frequency. In contrast, nonlinear analysis of the contraction motion assesses the signal on a point-by-point basis, and takes into account the entire structure of the signals.

We also demonstrated that this nonlinear analytical tool can supplement existing measures of contractile physiology, in order to more precisely characterize hiPSC-CM drug response. The key findings based on nonlinear analysis indicated that each drug produced unique effects on not only contractile frequency and amplitude, but also contractile dynamics by accounting for inter-beat arrhythmic behaviors reflected by their nonlinear characteristics. By comparing drug response of alfuzosin and isoproterenol, we found that though both drugs increased the beat rate of hiPSC-CMs (contractile frequency), but only isoproterenol enhanced the contraction/relaxation velocities (contractile amplitude). Such differences between two drugs led to the opposite changes in correlation dimension, shown as a decrease of correlation dimension with alfuzosin but an increase of correlation dimension with isoproterenol corresponding to increment dosage of each drug. However, although flecainide induced significant changes in contractile frequency, it did not influence significantly on the contractile dynamics determined by the nonlinear analysis. These results highlight that incorporation of nonlinear dimensional analysis into current available analytical toolboxes can be useful for quantifying the variability, complexity and irregular arrhythmic behavior in cardiac contractile dynamics in response to different drug exposure.

Arrhythmia classification in ECGs and cardiac signals is widely recognized as a critical measure for the diagnostics and management of heart-related disorders. Many traditional techniques, however, are limited in their processing capabilities. As described by Guitierrez-Gnecchi *et al*., digital signal processing algorithms were implemented to classify various heartbeat conditions^32^. ECGs have also been classified automatically using Support Vector Machines, which produced more favorable outcomes than other machine learning techniques^33^. Both techniques and many others, however, rely on singular analysis of inter-beat time intervals. As such, these methods require additional processing techniques, such as segmentation and feature extraction of the signals^34^. This not only extends the analytical time, but can also omit potentially critical signal characteristics. Although this research direction has promising potential for clinical application of beat-to-beat analysis for heart rate variability, conditions where severe arrhythmic activity and abnormalities are prominent throughout the entire signal will present challenging obstacles. Nonlinear analysis overcomes many of these challenges because of the ability to characterize a signal in its entirety and classify the complex dynamics exhibited.

In previous work applying nonlinear signal reconstruction to biological systems, cortical function at different sleep stages were quantified using EEG signals. This analysis illustrated that the entropy and correlation parameters of phase space plots are unique to each stage of sleep. In addition, nonlinear transforms of cardiac signals have been widely performed to characterize cardiology from ECG signals and assess heart rate variability (HRV)^35–37^. Patients prone to high risk of cardiac disease and mortality showed distinct heart rate dynamics, including decreased fractal organization^38^, based on nonlinear analysis of biological signals. Furthermore, evidence also strongly suggests that signals acquired from patients suffering with life-threatening conditions, such as disease, aging, and trauma, exhibit loss of multiscale complexity^38–41^. Since these changes are shown to be prominent under certain physiological conditions, nonlinear analysis has evolved to become an important clinical tool for clinicians and cardiologists.

The development of signal processing techniques in biomedical research remains a priority in order to diagnose and treat patients sooner. Because of its’ increasing recognition in clinical settings, we have extended phase space reconstruction signal analysis to *in vitro* cardiac model systems for characterizing the cardiac signals obtained from hiPSC-CMs. Implementation of optical-flow based motion tracking to hiPSC-CMs has allowed us to quantify the variations in contractile physiology, providing a linear time domain assessment of beat rate and contraction velocity. The essential effort of this work is to reconstruct these time series of experimental data into a multi-dimensional phase space for nonlinear analysis on the contractile dynamics of hiPSC-CMs, providing a quantitative measure of *in vitro* system complexities. We envision that this integrated approach can potentially be useful for health monitoring and diagnostics, such as assessment of heart rate variabilities and for early predictions of disease onset. In addition, the application of this analytical toolbox in pharmacological cardiotoxicity is essential to better understand drug effects on cardiac health that are not readily apparent and cannot be observed through time domain cardiac signals.

## Supplementary information

Supplementary Material

Supplemental Movie 1

Supplemental Movie 2

Supplemental Movie 3

Supplemental Movie 4
