# Supplementary material for: Quantification of Contractile Dynamic Complexities Exhibited by Human Stem Cell-Derived Cardiomyocytes Using Nonlinear Dimensional Analysis
Source: Sci Rep. 2019 Oct 11;9:14714. doi: 10.1038/s41598-019-51197-7 (PMC6789143; doi:10.1038/s41598-019-51197-7)
Supplement: Supplementary file 1 — Supplementary Material [file 41598_2019_51197_MOESM1_ESM.pdf]

Quantification of Contractile Dynamic Complexities Exhibited by Human Stem Cell-Derived Cardiomyocytes Using Nonlinear Dimensional Analysis

Plansky Hoang[1,2], Sabir Jacquir[3#], Stephanie Lemus[1,2], Zhen Ma[1,2#]

[1]Department of Biomedical and Chemical Engineering, Syracuse University, Syracuse, NY, USA
[2]Syracuse Biomaterials Institute, Syracuse University, Syracuse, NY, USA
[3]Department of Integrative and Computational Neuroscience, Paris-Saclay Institute of Neurosciences, Université Paris-Sud - Université Paris-Saclay, Cedex, France

[#]Correspondence:
Sabir Jacquir
Department of Integrative and Computational Neuroscience, Paris-Saclay Institute of Neurosciences, Université Paris-Sud - Université Paris-Saclay, Cedex, France
Email: sabir.jacquir@u-psud.fr

Zhen Ma
Department of Biomedical and Chemical Engineering, Syracuse University, Syracuse, NY, USA
Email: zma112@syr.edu

Supplementary Materials

Movie Captions

Supplemental Movie 1. Motion tracking vector calculation of contraction motion exhibited by *Cluster A*
Supplemental Movie 2. Motion tracking vector calculation of contraction motion exhibited by *Cluster B*
Supplemental Movie 3. Nonlinear phase space reconstruction of contraction motion exhibited by *Cluster A*
Supplemental Movie 4. Nonlinear phase space reconstruction of contraction motion exhibited by *Cluster B*

Supplemental Figure 1

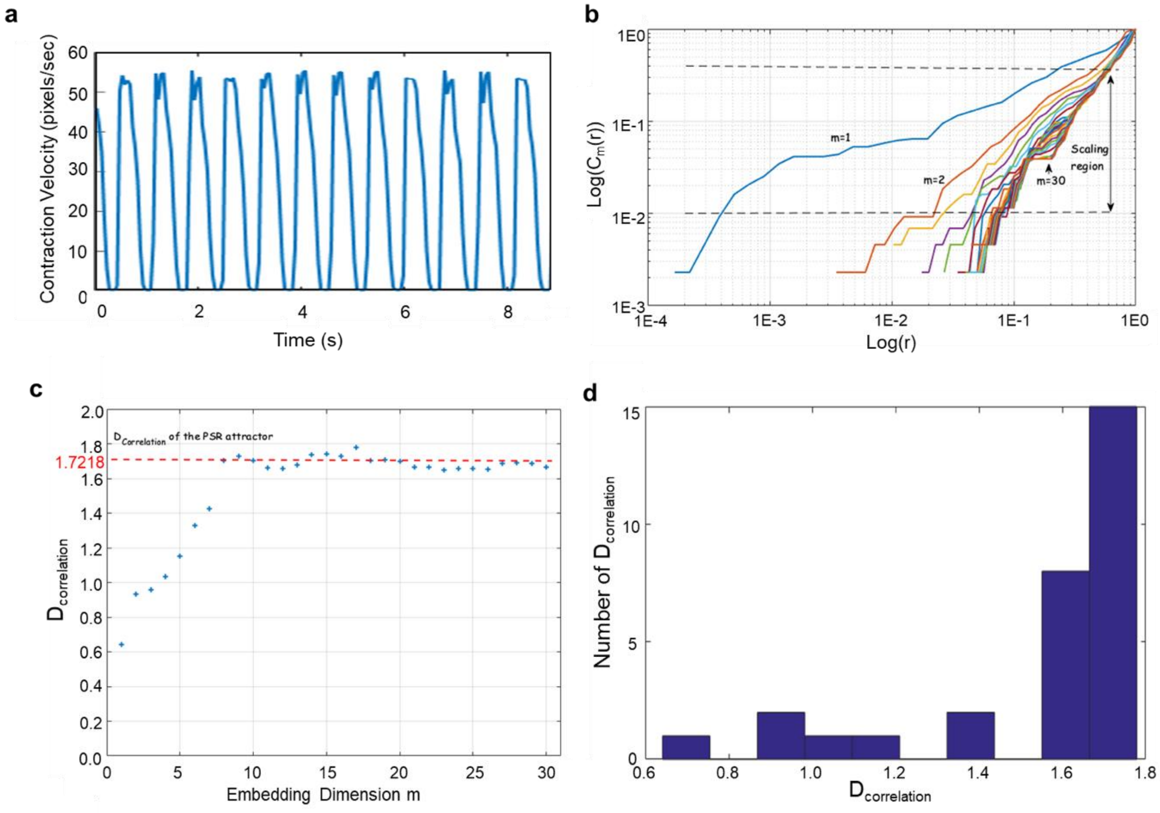

Supplemental Figure 1. Computation of capacity and correlation dimensions. (a) A representative contractile motion waveform is used to compute the capacity dimension and correlation dimension. (b) The correlation integral $C_{m(r)}$ of the motion waveform is plotted for each embedding dimension to demonstrate the scaling regions. Based on the correlation integral, (c) the correlation dimension can be computed with respect to the embedding dimension and (d) plotted as a histogram of the distribution.

Supplemental Figure 2

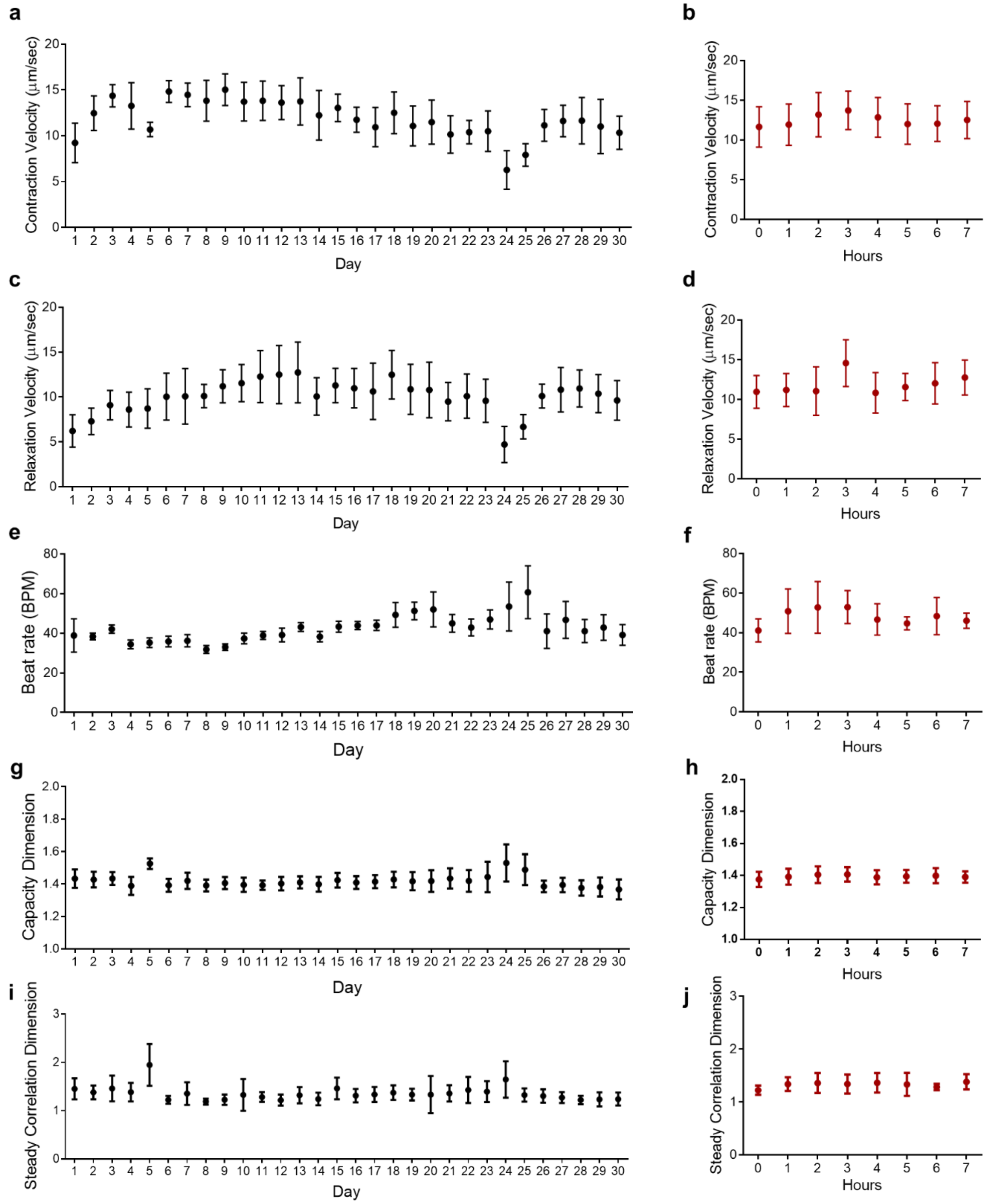

Supplemental Figure 2. Nonlinear analysis on contractile variability over daily and hourly time scales. The contractile behaviors of total six hiPSC-CMs samples were evaluated over a consecutive 30-day period and 7-hour period, including contraction velocities (a, b)**,** relaxation velocities (c, d) and beat rates (e, f). From nonlinear analysis of the motion waveforms, fluctuations and variance in the capacity dimension among six samples were measured (g) across the 30-day period, (h) but negligible changes were observed over the 7-hour period. (i, j) The steady correlation dimension was found with higher variance within the samples and more fluctuations across these time scales than capacity dimension

Supplemental Figure 3

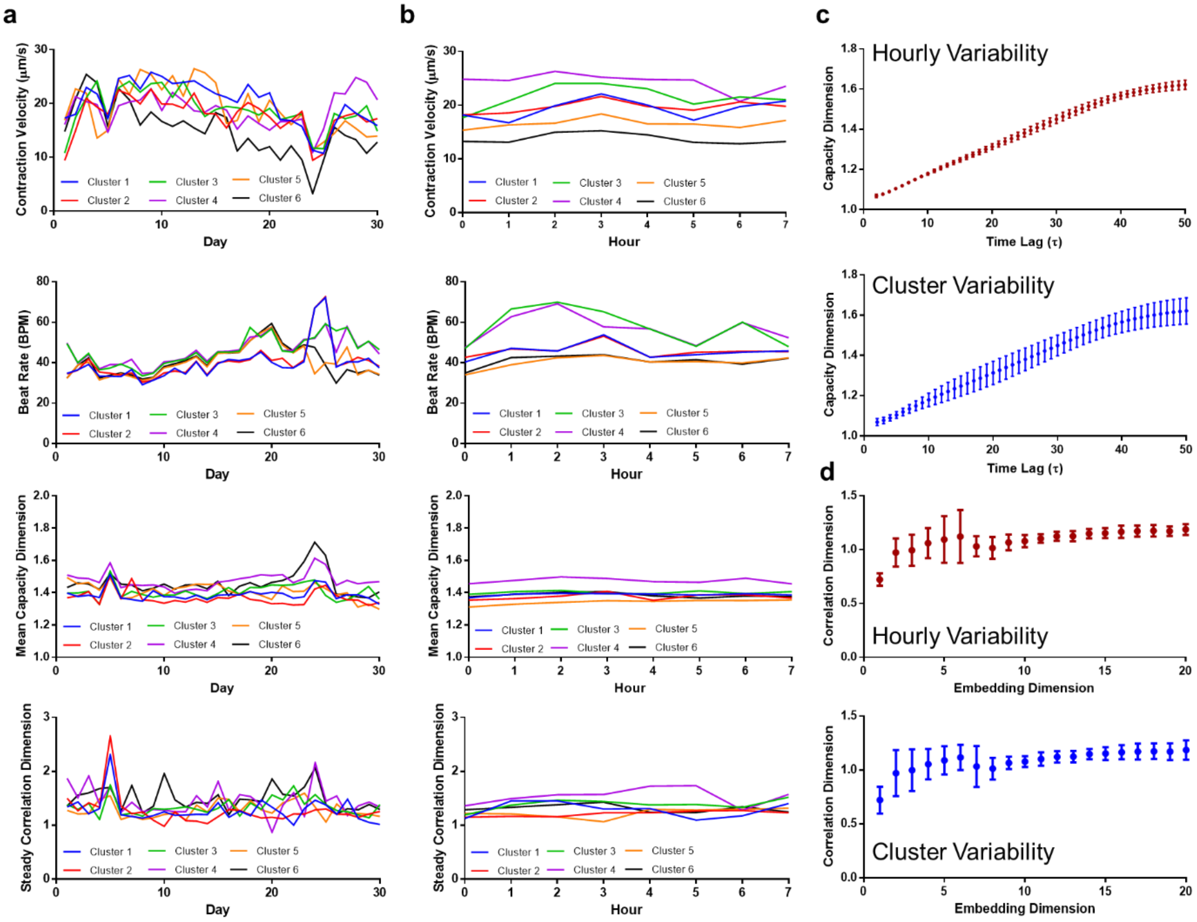

Supplemental Figure 3. Daily and hourly variability in contractile analysis and nonlinear dynamics analysis for individual hiPSC-CMs clusters. (a) Daily variability for each cluster was plotted in contraction velocity, beat rate, and capacity dimension, and steady correlation dimensions over a 30-day period. Similarly, (b) hourly variability for each cluster was plotted over a 7-hour period. (c, d) The capacity and correlation and dimensions over the hourly time study showed the higher standard deviations of cluster variability than hourly variability.

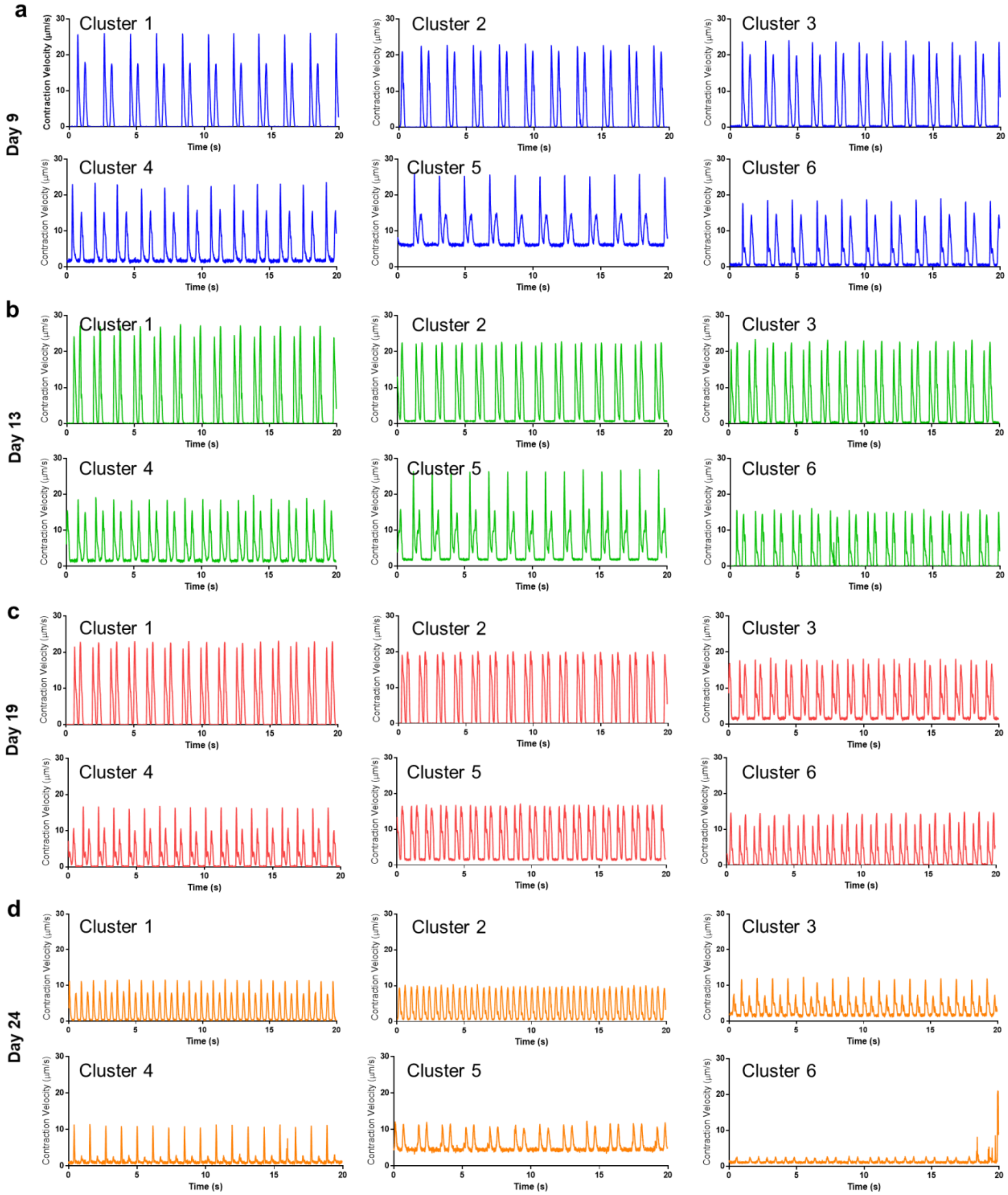

Supplemental Figure 4. Variability in contraction motion waveforms among six hiPSC-CM clusters across four selected days. Motion waveforms of individual tissue clusters plotted for Days (a) 9, (b) 13, (c) 19, and (d) 24.

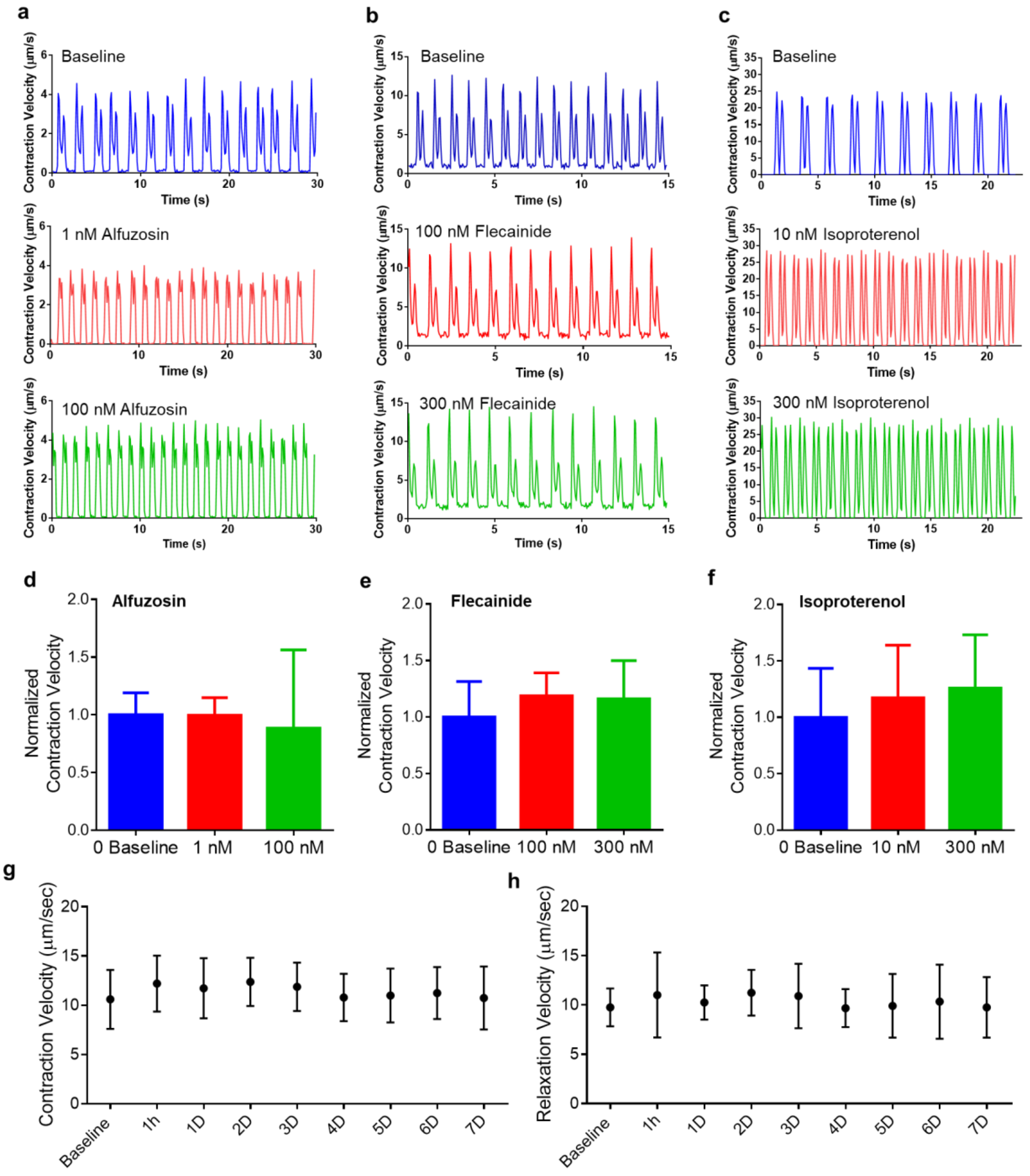

Supplemental Figure 5. Contractile velocities of hiPSC-CMs exposed to drug compounds. Short term changes to contraction motion waveform and average contraction velocities were observed in hiPSC-CMs treated (a, d) alfuzosin, (b, e) flecainide and (c, f) isoproterenol. (g, h) Long-term treatment of hiPSC-CMs with isoproterenol showed negligible changes in both contraction and relaxation velocities over a 7-day period.
